# UTILIZATION OF COMMUNITY CARE SERVICE AND SUBJECTIVE WELL-BEING OF OLDER PEOPLE: EVIDENCE FROM WEIFANG, CHINA

**DOI:** 10.1093/geroni/igad104.2903

**Published:** 2023-12-21

## Abstract

Many developed countries have established community-based care systems to address care needs of older people. However, community-based care in China is still in an early stage, some of its shortcomings are highlighted under the COVID-19 pandemic. Studying the wellbeing effect of community care in China could provide policy reference for developing countries. Based on the Elderly Health Survey in Weifang, China, using the two-stage least squares method, the effect and mechanism of the utilization of community care services on the subjective wellbeing of elder people were evaluated. Results show that: first, the utilization of community care services could promote the subjective wellbeing of older people and has a stronger effect on people with a higher level of subjective wellbeing. Second, the utilization of different types of community elderly care services has different effects on the subjective wellbeing of older people. When receiving a single type of community elderly care services, medical care services have the strongest promotion effect, while taking spiritual and cultural services and other types of services together will be a stronger synergistic effect on subjective wellbeing. Third, with the support of various community service subjects, the utilization of community care services could improve the subjective wellbeing by improving their health status, sleep quality and declining feeling of pain. In this regard, this study proposes policy suggestion in optimizing the types of community care service projects, building elderly care service consortiums, and improving community health management service capabilities to coping with deep aging and fewer birth.

